# Vitamin D and thyroid function of pregnant women in a sunny region: is there any connection?

**DOI:** 10.1590/1806-9282.20241028

**Published:** 2025-03-31

**Authors:** Marcos Oliveira Pires de Almeida, Lúcio Vilar, Alcides da Silva Diniz

**Affiliations:** 1 Brazilian Company of Hospital Services, Federal University of Pernambuco, Postgraduate Program in Nutrition – Recife (PE), Brazil.; 2 Federal University of Pernambuco, Hospital of Clinics, Division of Endocrinology – Recife (PE), Brazil.; 3 Federal University of Pernambuco, Postgraduate Program in Nutrition – Recife (PE), Brazil.

**Keywords:** Pregnancy, Thyroid hormones, Vitamin D, Tertiary care

## Abstract

**OBJECTIVE:**

The aim of this study was to investigate the relationship between vitamin D serum concentrations and thyroid hormones of pregnant women living in a sunny region of Northeast Brazil.

**METHODS:**

This is a cross-sectional secondary analysis of a study that evaluated the iodine status of pregnant women attending prenatal consultations in a tertiary hospital. Consecutive patients (>18 years) were conveniently sampled. Blood samples were collected for the study, including measurements of vitamin D, anti-TPO, and anti-Tg antibodies, thyroid-stimulating hormone, total and free thyroxine (TT4, FT4), and total and free triiodothyronine (TT3, FT3). Vitamin D levels <20 ng/mL were considered deficient. Between 20 and 30 ng/mL were classified as insufficient, and >30 ng/mL were sufficient. Additional data on urinary iodine concentrations, anthropometry, sociodemographic, and gestational features were also collected.

**RESULTS:**

A total of 562 pregnant women were included, with a median age of 29 years. Most of them (47.9%) were in the first gestational semesters. Only 3.9% of subjects presented with vitamin D deficiency, while 81% had insufficient values and 15.1% had sufficient levels. Vitamin D levels were not significantly correlated or associated with thyroid hormones or body mass index (all p-values >0.05).

**CONCLUSION:**

Our study adds to the growing body of evidence highlighting the importance of assessing the status of both vitamin D and thyroid hormones in population- and region-specific contexts, as it may vary accordingly.

## INTRODUCTION

Thyroid plays a role in human metabolism and other bodily functions^1,2^. During pregnancy, the importance of this relationship increases significantly^3^, as pregnancy promotes significant alterations in metabolism and hormonal status^3^. It is well-established that thyroid hormones are essential for the growth and development of the fetus^4,5^. Consequently, thyroid disorders during pregnancy can lead to significant health repercussions, including both short- and long-term events^6^. This relationship is particularly relevant during the first trimester, as the fetus relies solely on maternal thyroid hormones^7^. It has been documented that thyroid disorders may result in intrauterine growth restriction^8^, premature labor^5^, and low APGAR scores^8^.

In addition to thyroid hormones, vitamin D has been described as an important nutrient related to endocrine functions^9^, beyond its classical roles in calcium-phosphorus homeostasis^10^. Vitamin D has demonstrated potential as a mediator of immune response and antimicrobial activity, connected with autoimmune diseases and potentially influencing the human gut microbiota^11^. During pregnancy, it also plays a significant role, as its deficiency correlates with poor gestational outcomes, including preeclampsia, gestational diabetes mellitus, bacterial vaginosis, and an increased risk for surgical interventions during labor^12,13^.

Evidence has described a potential link between vitamin D and thyroid hormones^14^. This connection may occur because thyrocytes (thyroid cells) express significant amounts of vitamin D receptors (VDR)^14^. In fact, vitamin D deficiency has been linked to autoimmune thyroid disorders^15,16^, which can lead to outcomes similar to those seen with thyroid disorders in pregnancy^12,13^. Despite this potential link, the relationship between vitamin D status and thyroid health remains elusive and requires further exploration, particularly in specific scenarios and regions. Therefore, this study aimed to investigate the relationship between serum concentrations of vitamin D and thyroid health in a population of pregnant women in tertiary care.

## METHODS

### Study design and subjects

This is a cross-sectional study with pregnant women (≥18 years) residing in a coastal region of Northeast Brazil. Women previously diagnosed with and aware of thyroid disorders, using multivitamin supplements, or medications containing iodine in their formulation were excluded. This manuscript was reported in accordance with the STROBE statement and was approved by the Institutional Ethics Committee (number: 5.047.319).

### Covariates

Relevant data were collected through clinical interviews and medical records checks. Age, marital status, educational level, income, ethnicity, and lifestyle factors were recorded for analytical purposes. Salt intake was self-reported by the participants. Clinical variables pertaining to gynecological history were assessed, including previous gestational history, number of pregnancies, and comorbidities. Body (kg) and weight (m) were measured to calculate body mass index (BMI in kg/m^2^). Gestational trimesters were defined as follows: the first trimester spanned up to 13 weeks and 6 days; the second trimester extended from the 14th week to 27 weeks and 6 days; and the third trimester encompassed from the 28th week to 41 weeks and 6 days.

### Iodine concentrations

Urinary iodine concentrations (UICs) were used to assess iodine status. It was determined using the Sandell and Kolthoff^17^ technique, modified by Esteves^18^, following recommendations from the International Council for Control of Iodine Deficiency Disorders-World Health Organization (ICCIDD-WHO). UIC in μg/L was classified according to ICCIDD-WHO criteria for pregnant women: excessive (≥250 μg/L), adequate (≥150 and <250 μg/L), and deficient (<150 μg/L). Only those with adequate iodine status were included in this subanalysis.

### Blood samples

All patients underwent blood sample collection by a collaborating phlebotomist via peripheral venous puncture. The samples were deposited into tubes for whole blood or serum, transported in thermal boxes with ice packs, and stored at 4°C until analysis. Serum levels of thyroid-stimulating hormone (TSH), anti-TSH antibodies, total triiodothyronine (TT3), free triiodothyronine (FT3), total thyroxine (TT4), and free thyroxine (FT4) were determined by chemiluminescence. Thyroglobulin, anti-thyroid peroxidase (anti-TPO), and anti-thyroglobulin (anti-Tg) assays were determined by electrochemiluminescence. The biochemical and hormonal assays were conducted using the Modular PP—Roche/Hitachi equipment with reagents from Roche Diagnostics. The determination of 25(OH)D (vitamin D) was also conducted. Levels <20 ng/mL were considered deficient. Between 20 and 30 ng/mL were classified as insufficient, and >30 ng/mL were sufficient. All tests were performed at a private laboratory in Recife-PE, under the supervision of a collaborating biomedical scientist. For this study, all thyroid-related hormones were treated as continuous variables.

### Statistical analysis

Data were analyzed using R Studio, version 4.3.2. Shapiro-Wilk test was employed to evaluate the normality of continuous variables. They were described using mean±standard deviation (SD), or median and interquartile ranges (IQ), as appropriate. Categorical data were described as frequencies (n, %) and compared through Pearson's χ^2^ or Fisher's exact test. Kruskal-Wallis's test was employed to compare median groups in relation to their vitamin D status. Spearman's rho correlation test was employed to analyze non-normally distributed continuous variables, assessing correlation coefficients between vitamin D, BMI, and thyroid hormones. A stepwise forward linear regression analysis was conducted to check the independence of correlation. Statistical significance was set at p<0.05 for all analyses.

## RESULTS

This study initially screened 569 subjects. Following exclusions, a total of 562 pregnant women were included in this analysis. Only 15.1% of the sample had sufficient serum levels of vitamin D, while the majority exhibited insufficiency (81%). Deficient levels were found in only 3.9%. Table 1 presents detailed characteristics of the sample regarding sociodemographic and gestational features, stratified by vitamin D status. In terms of sociodemographic and lifestyle aspects, no differences were observed between groups based on vitamin D concentrations. No significant differences were also observed.

**Table 1:** Characteristics of pregnant women in relation to vitamin D status (n=562).

Variables		<20 ng/mL	20–30 ng/mL	>30 ng/mL	p-value
n, %		22 (3.9)	455 (81.0)	85 (15.1)	
Sociodemographic features					
	Age (years)	28 (27–30)	29 (27–33)	30 (27–32)	0.72^a^
Skin color					
	White	6 (27.3)	156 (34.3)	24 (28.2)	0.46^b^
	Non-white	16 (72.7)	299 (65.7)	61 (71.8)	
Educational level					
	Primary	12 (54.5)	212 (46.6)	36 (42.4)	0.52^b^
	Secondary	7 (31.8)	211 (46.4)	43 (50.6)	
	Superior	3 (13.6)	32 (7.0)	6 (7.1)	
Marital status					
	No partner	4 (18.2)	48 (10.5)	9 (10.6)	0.53^b^
	With partner	18 (81.8)	407 (89.5)	76 (89.4)	
Income					
	<1 minimum wage	3 (13.6)	75 (16.5)	13 (15.3)	0.80^b^
	1–2 minimum wages	9 (40.9)	229 (50.3)	41 (48.2)	
	>2 minimum wages	10 (45.5)	151 (33.2)	31 (36.5)	
Smoking		1 (4.5)	24 (5.3)	8 (9.4)	0.32^b^
Alcoholism		4 (18.2)	82 (18.0)	17 (20.2)	0.91^b^
Gestational features					
Trimester					
	First	14 (63.6)	218 (47.9)	37 (43.5)	0.34^b^
	Second	2 (9.1)	124 (27.3)	24 (28.2)	
	Third	6 (27.3)	113 (24.8)	24 (28.2)	
High-risk pregnancy		3 (13.6)	110 (24.2)	19 (22.4)	0.50^b^
N° of carriages					
	One live birth	4 (18.2)	98 (21.5)	16 (18.8)	0.95^b^
	Two live births	3 (13.6)	72 (15.8)	13 (15.3)	
	Three live births	3 (13.6)	34 (7.5)	6 (7.1)	
Previous miscarriage		3 (13.6)	38 (8.4)	1 (1.2)	*
Premature labor history		3 (13.6)	25 (5.5)	2 (2.4)	*
Salt intake					
	None	3 (13.6)	16 (3.5)	5 (5.9)	0.16^b^
	Only salt	15 (68.2)	314 (69.0)	55 (64.7)	
	Salt+industrialized seasoning	4 (18.2)	125 (27.5)	25 (29.4)	
Iodine status					
	Sufficient	4 (18.2)	106 (23.3)	14 (16.5)	0.34^b^
	Insufficient	18 (81.8)	349 (76.4)	71 (83.5)	
Comorbidities					
	Systemic arterial hypertension	0 (0.0)	10 (2.2)	4 (4.7)	*
	Type 2 diabetes mellitus	1 (4.5)	5 (1.1)	1 (1.2)	
	Preeclampsia	0 (0.0)	10 (2.2)	4 (4.7)	
	Gestational diabetes mellitus	2 (9.1)	46 (10.1)	10 (11.8)	

a Kruskal-Wallis test (median and interquartile range);

b Pearson's χ^2^ or Fisher's exact test (frequencies, n and %);

* Absence of p-value due to very low sample size.

When comparing median values of nutritional features (BMI and UIC concentrations) along with thyroid function hormones, no differences were observed between groups in relation to their vitamin D status (Table 2). No significant correlation was observed between serum vitamin D concentrations and nutritional features or thyroid hormones (Table 3). The linear regression analysis model, utilizing vitamin D as the dependent variable and employing a stepwise forward model, did not include any nutritional or thyroid hormones in the model. Consequently, they did not significantly explain the variability of vitamin D serum concentrations.

**Table 2:** Kruskal-Wallis’ test: Median comparisons of nutritional status and thyroid hormones among pregnant women in relation to serum concentrations of vitamin D (n=562).

Variables	<20 ng/mL	20–30 ng/mL	>30 ng/mL	p-value
BMI (kg/m^2^)	29.5 (25.1; 32.2)	27.2 (23.1; 32.3)	27.2 (23.9; 32.5)	0.80
UIC	159.8 (155.1; 166.4)	157.4 (150.7; 166.1)	157.7 (151.9; 164.9)	0.58
Anti-TPO	19.3 (12.9; 27.7)	20.0 (10.9; 28.2)	22.9 (13.0; 29.3)	0.30
Anti-TSH	0.62 (0.44; 0.84)	0.73 (0.51; 0.98)	0.71 (0.51; 0.89)	0.17
Tg-Ab	131.3 (127.2; 139.2)	130.2 (125.9; 135.2)	130.3 (126.5; 137.2)	0.77
Tg	15.2 (13.7; 16.7)	15.7 (14.7; 17.3)	15.7 (14.7; 17.7)	0.24
TSH	1.81 (1.08; 2.58)	2.01 (1.16; 2.46)	1.84 (1.04; 2.31)	0.33
F_T4_	1.1 (1.0; 1.3)	1.0 (0.9; 1.2)	1.0 (0.9; 1.1)	0.12
T_T4_	11.8 (11.0; 12.6)	11.4 (11.0; 11.9)	11.4 (11.0; 12.0)	0.52
F_T3_	0.31 (0.31; 0.34)	0.30 (0.26; 0.35)	0.30 (0.26; 0.35)	0.35
T_T3_	131.3 (127.2; 139.2)	130.2 (125.9; 135.2)	130.3 (126.5; 137.2)	0.62
T3/T4 ratio	11.26 (10.32; 12.57)	11.47 (10.77; 12.22)	11.38 (10.66; 12.17)	0.77

Anti-TPO: thyroid peroxidase antibody; BMI: body mass index; FT3: free triiodothyronine; FT4: free thyroxine; Tg-Ab: thyroglobulin antibody; TSH: thyroid-stimulating hormone; TT3: total triiodothyronine; TT4: total thyroxine; UIC: urinary iodine concentration.

**Table 3:** Spearman's (ρ/rho) correlations: correlation coefficients between serum concentrations of vitamin D, nutritional status, and thyroid hormones among pregnant women (n=562).

Variables	Coefficient	p-value
BMI (kg/m^2^)	-0.01	0.79
UIC	-0.03	0.50
Anti-TPO	0.02	0.63
Anti-TSH	0.01	0.98
Tg-Ab	-0.01	0.77
Tg	0.08	0.06
TSH	0.01	0.88
F_T4_	0.07	0.12
T_T4_	0.01	0.93
F_T3_	0.06	0.19
T_T3_	0.03	0.53
T3/T4 ratio	0.03	0.45

Anti-TPO: thyroid peroxidase antibody; BMI: body mass index; FT3: free triiodothyronine; FT4: free thyroxine; Tg-Ab: thyroglobulin antibody; TSH: thyroid-stimulating hormone; TT3: total triiodothyronine; TT4: total thyroxine; UIC: urinary iodine concentrations.

## DISCUSSION

Our main findings indicated no significant differences in vitamin D status when considering various sociodemographic and gestational features. Furthermore, no correlation or association was observed between vitamin D levels and thyroid-related markers. A previous study conducted in China found contrasting results^19^. The authors demonstrated an independent association between vitamin D levels and TSH, as well as F_T3_ and F_T4_. They also observed that in the first gestational trimester, F_T3_ levels increased progressively with higher concentrations of vitamin D. In contrast, similar to our findings, a study conducted in Iran involving pregnant women found no significant correlations between vitamin D levels and thyroid hormones^20^. These inconsistencies and discrepancies may be explained by several factors that influence both vitamin D levels and thyroid function. It includes ethnicity, geographic region, exposure to sunlight, dietary intake, sociodemographic and economic aspects, supplement use, and individual biological differences.

These contrasting results raise critical queries about whether there is a genuine connection between vitamin D status and thyroid hormones, despite their modest link through shared receptors^14^. In this context, there remains a significant gap in understanding this potential relationship and the underlying mechanisms, which is crucial for improved comprehension and clinical interventions. However, exploring this relationship in specific populations and regions contributes to the growing body of evidence demonstrating the potential influence of environmental and individual regional factors on human bodily functions and health^21^.

Our additional findings demonstrated a relatively low frequency of vitamin D deficiency, which contrasts with a previous study conducted in a different region of Brazil (southern)^22^. While we found a deficiency rate of 3.9%, the authors of the southern study reported a rate of 43.7%^22^. This discrepancy reinforces the influence of latitude and region-specific characteristics on serum concentrations of this vitamin. The authors of the southern study indicated a close link between vitamin D levels and season, with deficiency being more evident during winter^22^. In the northeast, our region of interest, cold weather is uncommon, and solar exposure is prevalent throughout the year in many areas^23^, potentially explaining these differences.

In contrast to our findings, the authors additionally^22^ reported a lower rate of insufficient vitamin D status (37.1%), while we found 81% in this classification. These findings raise additional insights, as there are ongoing debates about what constitutes insufficient vitamin D status. The authors discuss the biases related to this classification, which, despite being endorsed by experts in clinical guidelines, are commonly based on cross-sectional and longitudinal observational studies rather than more robust designs^24^. They also discuss the difficulty of achieving optimal vitamin D targets (>30 ng/mL), suggesting a potential insufficiency or deficiency rate of 90% in the human population^24^.

It is relevant to discuss that in Brazil, vitamin D supplementation during pregnancy is endorsed by a Ministry of Health policy^25^, as it potentially improves maternal and fetal health outcomes^26^. This endorsement aligns with ongoing debates about the current recommended targets for vitamin D concentrations^24^, suggesting that all pregnant women might receive adequate doses of vitamin D supplementation. However, future studies could further investigate these aspects to provide more comprehensive insights about vitamin D supplementation during pregnancy and proposed targets.

Although our study did not find a significant correlation between vitamin D levels and thyroid hormones, the high prevalence of vitamin D insufficiency (81%) in our cohort underscores the need for greater attention to this nutrient among pregnant women, even in regions with abundant sunlight. The implications for public health policies, particularly those focused on prenatal care, are significant. As previously highlighted^12,13^, vitamin D is essential for maternal and fetal health, playing critical roles in bone development and immune function, and potentially influencing pregnancy outcomes. While there remains a gap in our understanding of the impact of insufficient, but not deficient, vitamin D status on pregnant women, this finding emphasizes the importance of routine screening and supplementation, when necessary, as recommended by the Brazilian Ministry of Health^25^. Proactive prevention through supplementation is often more effective and feasible than treating deficiencies once they arise.

The discrepancies observed between our study and others^22,23^ may be attributed to biological and behavioral differences among populations. Factors such as BMI, age, race/ethnicity, and lifestyle behaviors, including outdoor activity and vitamin D supplementation, likely influence both vitamin D status and its potential effect on thyroid function. Notably, a previous study^22^ explored an additional factor—skin phototype—which we were unable to assess in our cohort. This is a critical aspect for future research, as individuals with darker skin may require longer sunlight exposure to synthesize adequate levels of vitamin D^22^. Thus, variations in phototype may partially explain differences in vitamin D insufficiency and deficiency across our populations.

It is also possible that differences in body mass phenotypes contribute to the discrepancies, as the previous study reported an initial mean BMI of 30 kg/m^2^, whereas in our cohort, higher BMI was more frequently observed among patients with vitamin D deficiency, despite no statistical difference being observed. Elevated BMI can influence vitamin D metabolism by increasing its sequestration in adipose tissue, potentially reducing serum concentrations and impacting study outcomes^24^.

The definition of optimal vitamin D levels during pregnancy remains a contentious and debatable issue. While current guidelines recommend concentrations above 30 ng/mL^24^, there is ongoing debate about whether these targets are sufficient to ensure the best obstetric and fetal outcomes. Although the certainty of evidence is still limited, growing research indicates that higher levels (40–60 ng/mL) may further enhance health benefits^27^. However, before establishing universally applicable thresholds, it is essential to consider various factors, including regional, ethnic, and behavioral differences in vitamin D synthesis and metabolism.

Our study has limitations that warrant acknowledgment. The relatively small sample size, single-center setting, and observational cross-sectional design preclude the establishment of causal relationships and limit the generalizability to broader populations of pregnant women. Additionally, unmeasured factors such as sunscreen use and dietary intake might potentially skew our findings, necessitating caution in their interpretation. On a positive note, our study provides valuable insights by focusing on a novel region and a specific population of pregnant women.

## CONCLUSION

This study adds to the growing body of evidence examining the relationship between vitamin D status and thyroid health among pregnant women living in a sunny region of Northeast Brazil. Our findings demonstrate no significant correlation or association between vitamin D levels and thyroid hormones. This suggests that the relationship might be heavily influenced by region-specific and environmental factors, although further investigation is needed to confirm these influences.
